# Procedural Success Prediction Scoring Systems Used in Percutaneous Coronary Interventions for Chronic Total Occlusions: A Systematic Evaluation

**DOI:** 10.3390/healthcare9081033

**Published:** 2021-08-11

**Authors:** Crischentian Brinza, Iolanda Valentina Popa, Alexandru Basarab, Radu Crisan-Dabija, Alexandru Burlacu

**Affiliations:** 1 Department of Interventional Cardiology, Cardiovascular Diseases Institute, 700503 Iasi, Romania; crischentian-branza@email.umfiasi.ro (C.B.); alexandru-florin-basarab@email.umfiasi.ro (A.B.); alexandru.burlacu@umfiasi.ro (A.B.); 2 Department of Internal Medicine, Faculty of Medicine, University of Medicine and Pharmacy “Grigore T Popa”, 700115 Iasi, Romania; radu.dabija@umfiasi.ro; 3 Pulmonology Department, Clinic of Pulmonary Diseases, 700115 Iasi, Romania; 4 Romanian Academy of Medical Sciences, 030167 Bucharest, Romania

**Keywords:** coronary total occlusion, percutaneous coronary intervention, optimal medical therapy, predictive scores, predictive clinical models

## Abstract

(1) Background: Data suggest that patients with coronary chronic total occlusion (CTO) managed with percutaneous coronary intervention (PCI) could have better outcomes than those treated with optimal medical therapy alone. We aimed to systematically review dedicated scoring systems used to predict successful PCI in patients with CTO. (2) Methods: Electronic databases of MEDLINE (PubMed), Embase, and Cochrane were searched. (3) Results: 32 studies were included. We provided insights into all available predictive models of PCI success in CTO including predictive performance, validations, and comparisons between different scores and models’ limitations. Considering the differences in the population included, coronary lesions, and techniques applied across clinical studies, the most used scores displayed a modest to good predictive value, as follows: J-CTO (AUC, 0.55–0.868), PROGRESS-CTO (AUC, 0.557–0.788), CL (AUC, 0.624–0.800), CASTLE (AUC, 0.633–0.68), and KCCT (AUC, 0.703–0.776). As PCI for CTO is one of the most complex interventions, using dedicated scoring systems could ensure an adequate case selection as well as preparation for an appropriate recanalization technique in order to increase chances of successful procedure. (4) Conclusion: Clinical models appear to be valuable tools for the prediction of PCI success in CTO patients. Clinicians should be aware of the limitations of each model and should be able to correctly select the most appropriate score according to real-life case particularities such as lesion complexity and operator experience in order to maximize success and achieve the best patients’ outcomes.

## 1. Introduction

Coronary chronic total occlusion (CTO) defines patients with complete coronary artery obstruction for at least 3 months [1]. Individuals with CTO are relatively frequent in a contemporary setting. In a nationwide registry from the Netherlands, 6.3% (*n* = 8343) of percutaneous coronary interventions (PCI) were performed for CTO [2]. More than a quarter of patients had CTO in one study, while PCI for CTO was performed in only 8.1% of cases [3]. Moreover, in patients with documented coronary artery disease (CAD), CTO could be a usual finding, as one study revealed a prevalence of 46% [4].

Despite the fact that the rate of PCI performed for CTO is low, the proportion of patients is continuously rising. In addition, CTO is associated with adverse clinical outcomes. One study that included patients with non-ST-segment elevation myocardial infarction observed that 12-month mortality was more significant in the group with CTO than in patients without CTO (21.1% and 11.9%) [5]. Patients with ST-segment elevation myocardial infarction and CTO also had a greater mortality risk during the 3 years of follow-up [6].

In the last decades, the scientific community paid great importance to CTO management. Whether CTO patients’ referral to PCI could have beneficial clinical implications was mainly investigated in the literature. According to the Task Force on myocardial revascularization of the European Society of Cardiology and European Association for Cardio-Thoracic Surgery, PCI should be considered as a therapeutic option for patients with CTO and resistant angina or documented ischemia compatible with the territory of the occluded coronary artery (class IIa recommendation, level of evidence B) [7]. Moreover, to improve heart failure symptoms, PCI could be considered in patients with CTO and low left ventricular ejection fraction if myocardial viability is demonstrated using non-invasive tests [1].

In a recent study involving patients with CTO of the proximal or middle left anterior descending artery, PCI was associated with a significantly lower cardiac death rate than optimal medical therapy (OMT) alone [8]. A meta-analysis including 39,771 patients observed that PCI for CTO might be linked to lower cardiovascular mortality, with similar major adverse cardiac events (MACE) incidence [9]. Patients with CTO who underwent PCI exhibited a lower MACE incidence, lower all-cause mortality, and cardiac death rate than the OMT-only group, as reported in another meta-analysis [10]. All these data suggest that patients with CTO managed invasively could have better outcomes than those treated with OMT alone.

The success rate represents a problem associated with CTO percutaneous recanalization due to procedural and anatomical difficulties. The procedural success rate of PCI varies across studies, from 61 to 99% [10]. A global expert consensus recommends four PCI strategies: antegrade wire escalation, antegrade dissection, retrograde wire escalation, and retrograde dissection. In addition, microcatheters and intravascular imaging could be used for PCI optimization [11].

Different clinical models were developed to estimate the probability of successful PCI in patients with CTO. One of the most used scores is the Japanese Multicenter CTO Registry (J-CTO). J-CTO score was integrated into a novel algorithm for treating CTO, which could help to select an anterograde or retrograde approach based on lesion difficulty evaluation [12]. Lesion assessment using dedicated scores before PCI is helpful for the appropriate selection of recanalization technique, which is the cornerstone of procedural success.

In light of the above mentioned, we aimed to (1) systematically review the scientific literature and assess the predictive power of all the reported clinical models used to estimate the probability of successful PCI in patients with CTO and (2) emphasize the importance of acknowledging all available predictive clinical models and correctly selecting the most appropriate one for each particular clinical setting in order to maximize success and achieve the best patients’ outcomes.

## 2. Materials and Methods

Preferred Reporting Items for Systematic Review and Meta-Analysis (PRISMA) checklist was used in the conduction process of the present systematic review [13].

### 2.1. Data Sources and Search Strategy

A literature search was performed in MEDLINE (PubMed), Embase, and Cochrane library databases from inception to April 2021, without time interval or language restrictions (Table S1). Additionally, Google Scholar and references from the cited publications were examined to find eligible studies. We also screened a registry of clinical trials (ClinicalTrials.gov accessed on 30 April 2021) to detect supplementary data. According to the PRISMA search checklist, the search strategy for all databases was illustrated in Table S1. In addition, we restricted the search to studies involving humans in the case of MEDLINE and Embase databases. The search implied the following MeSH terms and keywords: “coronary chronic total occlusion”, “percutaneous coronary intervention”, “prediction”, “score”, “Japanese chronic total occlusion score (J-CTO)”, “prospective global registry for the study of chronic total occlusion intervention score (PROGRESS-CTO)”, “coronary artery bypass grafting (CABG) history, age (≥70 years), stump anatomy, tortuosity degree, length of occlusion and extent of calcification score (CASTLE)”, “clinical and lesion-related score (CL)”, “ostial location, Rentrop grade, age score (ORA)”, “coronary CT angiograph-derived registry of CrossBoss and hybrid procedures in France, the Netherlands, Belgium, and United Kingdom score (RECHARGE)”, “computed tomography registry of chronic total occlusion revascularization score (CT-RECTOR)”, and “Korean multicenter CTO CT registry score (KCCT).”

### 2.2. Eligibility Criteria and Outcomes

Studies retrieved after searching in databases were sought for inclusion if they fulfilled the prespecified eligibility criteria in concordance with the PICO checklist: (1) adult humans aged ≥18 years with CTO were enrolled; (2) original data were reported regarding scores for PCI success prediction in the case of CTO; (3) predictive performance of a particular score was reported; (4) studies that developed or validated internally or externally a clinical model; (5) studies that involved a comparison between different scores-when available; (6) outcomes of interest were recorded: the predictive power of available scores for the technical or procedural success of PCI performed for CTO. Also, we prespecified several critical exclusion criteria: unpublished data, inability to extract data, studies available only in abstract, case reports, letters, editorials, and meta-analyses. Two independent investigators established if studies fulfilled the inclusion criteria, and disagreements were solved by consensus.

### 2.3. Data Collection

The following data were extracted from included studies in the present systematic review by two independent investigators: first author, year, study design, number of patients included and their age, setting, rate of successful PCI, scores evaluated, and their predictive performance. Whenever possible, data were presented as numbers, percentages, median or mean values, c-statistic/area under the curve (AUC) with the corresponding 95% confidence interval and *p*-value. Discrepancies that appeared in the data collection process were solved by consensus.

### 2.4. Risk of Bias

The risk of bias and applicability of studies included in our systematic review were evaluated using the Prediction model Risk of Bias ASsessment Tool (PROBAST), designed for prediction model development and validation studies [14]. Briefly, the PROBAST tool encompasses four domains (participants, predictors, outcome, and analysis) with 20 signaling questions that guide the overall risk of bias and concerns regarding applicability estimation.

## 3. Results

**Figure 1:**
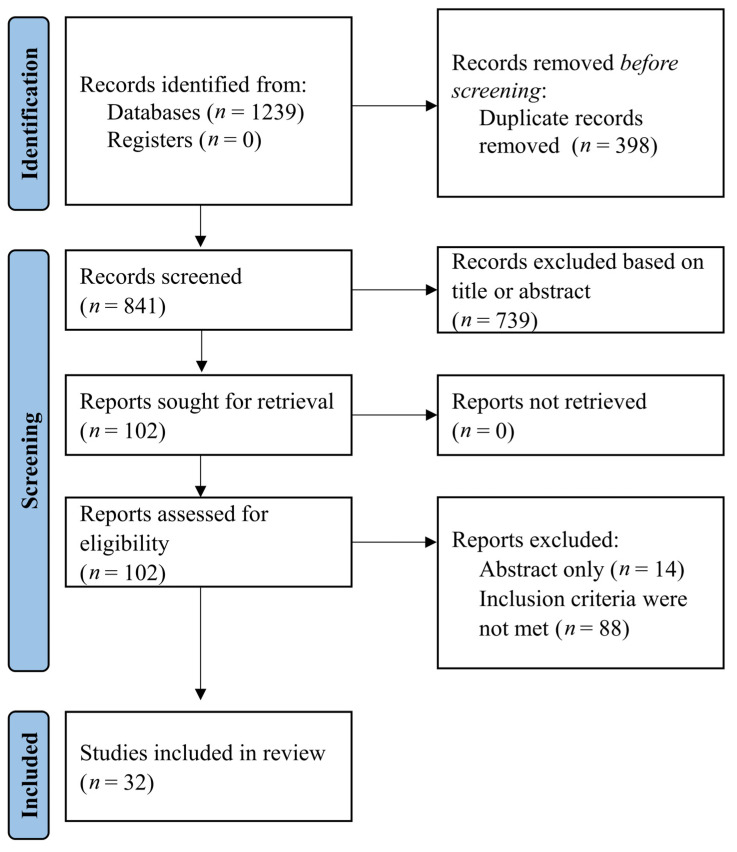
PRISMA Flow diagram for study selection.

Our search in the prespecified databases retrieved 1239 references. Initially, duplicate citations and records based on title or abstract were excluded, leaving 102 studies for eligibility assessment. Finally, 32 studies were included in the present systematic review, excluding 14 manuscripts available only in abstract and 56 citations that did not fulfill the inclusion criteria. The flow diagram of the search process is presented in Figure 1, in concordance with PRISMA criteria.

Data regarding studies’ general characteristics, design, population, clinical setting, and outcomes are presented in Table 1. Variables included in the most used predictive scores for successful PCI are provided in Table S3.

All included studies [15,16,17,18,19,20,21,22,23,24,25,26,27,28,29,30,31,32,33,34,35,36,37,38,39,40,41,42,43,44,45,46] had an observational design with the scores being applied retrospectively. The most investigated score in clinical studies was J-CTO [15,17,20,21,22,23,24,25,26,27,28,29,30,34,37,38,40,41,42,43,44,45,46] followed by PROGRESS-CTO score [18,22,24,28,37,38,41,44,46]. PCI success rate varied across studies from 48.2% [30] to 94.4% [21]. Results regarding different scores’ prediction power reported in clinical studies are presented in Table 2.

J-CTO score was initially derived from a cohort of 329 patients and validated in 165 patients [30]. Despite the low PCI rate defined as guidewire crossing within 30 min (48.2%), the J-CTO score showed good discrimination power in the derivation cohort (AUC 0.82), which was slightly lower in the validation cohort (AUC 0.76). However, the prediction power of the J-CTO score was not consistent in all studies. A lower performance (AUC 0.55) was reported by Ellis et al. [19] in the hybrid approach PCI. At the same time, the highest predictive ability (AUC 0.868) was observed by Li et al. [26]. Interestingly, in more complex lesions, the J-CTO score performed worse in recanalization prediction (AUC 0.473) [23]. Regarding PCI strategy, the J-CTO score had an excellent prediction value for antegrade procedures techniques (AUC 0.735). [24] In addition to the angiography-derived score, a less invasive computed tomography (CT)-derived J-CTO score could be a valuable tool for lesion stratification, with AUC ranging from 0.673 [27] to 0.882 [26], even in the context of a low PCI success rate (52.7%).

PROGRESS-CTO score derivation cohort consisted of 521 patients and was subsequently validated internally in 260 patients with a high PCI success rate (92.9%) [41]. In the derivation cohort, the predictive performance was higher (AUC 0.778) than in other studies that externally validated the score, with AUC ranging from 0.557 [37] to 0.788 [38] in the case of elderly patients.

Three dedicated CT-derived scores (RECHARGE, CT-RECTOR, KCCT) were developed to predict PCI success. In the initial derivation cohort (590 patients), the angiography-derived RECHARGE score performed better than J-CTO and PROGRESS-CTO scores in successful recanalization for CTO (respectively, AUC 0.783 vs. 0.676 and 0.608) [28]. Another study included in the present systematic review noticed a similar prediction value for procedural success and a 30-min wire crossing in the case of both CT-derived and angiography-derived RECHARGE scores [27].

CT-RECTOR clinical model was developed from a study involving 229 patients with CTO and better prediction power than the J-CTO score for guidewire crossing within 30 min (AUC 0.83 vs. 0.71) [34]. CT-RECTOR score maintained its superiority compared with J-CTO score for guidewire crossing in 30 min and final procedural success prediction in another two studies [43,44]. However, one study showed discrepant results, as the J-CTO score had greater performance than the CT-RECTOR score (respectively, AUC 0.704 vs. 0.665) [27].

KCCT score was developed by Yu et al. [44]. It was externally validated by Li et al. [27]. KCCT prediction model displayed excellent discriminatory power for guidewire crossing in 30 min (AUC 0.776 vs. 0.703, respectively) and final procedural success (AUC 0.773 vs. 0.717, respectively), which was consistent in both studies. Roller et al. [36] proposed a new five-item CT-based score with good prediction power (AUC 0.8232). Nonetheless, a small cohort of patients was included, and results should be confirmed in more extensive studies.

CASTLE (EuroCTO) score was derived and externally validated in large cohorts of patients [22,23,37,39]. Though the CASTLE score had a modest predictive value, with AUC ranging from 0.633–0.68, it was comparable to the J-CTO score. The CASTLE score had lower performance in complex lesions, but it was still better than the J-CTO score (AUC 0.588 vs. 0.473). [23]

Since its development, the CL score has been extensively validated in different studies [15,22,24,37,38,44]. The predictive value of the CL score varies across studies with AUC ranging from 0.624 [22] to 0.800 [38], which is slightly better than the J-CTO score in some cases [15,24,37].

Some studies developed original scores for successful PCI prediction [16,19,25,31,32,33,35]. J-Channel score had good discriminatory power for septal and non-septal collateral channel guidewire crossing success in the case of PCI by retrograde approach (respectively, an AUC of 0.744 and 0.757 in the derivation cohort) [31]. Recently developed and validated in-stent chronic total occlusion (IS-CTO) score had an exceptional predictive power (AUC 0.976) for antegrade PCI in the case of in-stent chronic total occlusion, which was significantly higher than the predictive value of J-CTO score (AUC 0.642) and PROGRESS-CTO score (AUC 0.579) [46].

The risk of bias and concern regarding applicability was determined using the PROBAST tool, [14], especially designed for clinical models’ development and validation studies. In general, the risk of bias was high, as all studies were observational and all scores were applied retrospectively (Table S2). There was a common concern regarding the applicability, as population, clinical setting, and outcomes were in concordance with objectives and inclusion criteria of the present systematic review.

## 4. Discussion

Our paper contributes to the literature with the first systematic review of all reported clinical models used to predict PCI success in patients with CTO. We have shown both sides of the coin: Firstly, PCI could have better outcomes than OMT alone in CTO patients, and, secondly, the CTO percutaneous recanalization may sometimes fail due to procedural and anatomical difficulties. Thus, we emphasized the importance of correctly predicting the most suitable recanalization technique and subsequent PCI success in order to optimize high-risk clinical decisions involving invasive versus OMT therapeutic strategies. Until the ideal predictive score is discovered, it is mandatory that clinicians and decision-makers have insight into all available predictive models of PCI success in CTO, are aware of their limitations, and are able to correctly select the most appropriate score according to real-life case particularities such as lesion complexity and operator experience. Thereby, the rigorous systematization of all validated clinical scores appeared as a necessity.

Recanalization techniques for CTO changed significantly over time. A hybrid algorithm involving both antegrade and retrograde approaches gained more evidence in the last years in terms of safety and efficacy [47]. A well-described lesion (including proximal and distal cap, occlusion length, calcification, bending, collateral vessels) represents the cornerstone of preparation for PCI. The probability of successful PCI could guide the choice of a recanalization technique, selecting guidewires and catheters to improve the final procedural outcome.

One of the most used scores in clinical practice is represented by the J-CTO score, which was initially derived and validated in a Japanese cohort of patients [30]. Five variables were included in the final prediction model: entry shape, calcification, bending, occlusion length, and previous failed PCI. For each variable, a maximum of 1 point could be assigned, and the lesion difficulty is stratified into four categories: easy (0 points), intermediate (1 point), difficult (2 points), and very difficult (≥3 points). The probability of guidewire crossing within 30 min was lower once the J-CTO score was higher (87.7%, 67.1%, 42.4%, 10.0%).

However, the ability of the J-CTO score to predict successful PCI was lower in the case of the hybrid approach or patients with more complex lesions, as documented by some studies [19,23,28]. J-CTO score had a modest to good predictive power for the procedural outcome and could also be a valuable tool for procedural time estimation. Taken together, PCI success and time estimation in addition to lesion difficulty grading could help in optimal therapeutic decision making: surgical or percutaneous myocardial revascularization versus OMT alone. The clinical applicability of the J-CTO score could be extended by a CT-derived version, with a similar or even better predictive value [20].

PROGRESS-CTO constitutes a promising score, which was validated in large cohorts of patients. It is also characterized by simple clinical applicability. Only four variables were included in the final prediction model: proximal cap ambiguity, absence of interventional collaterals, moderate/severe tortuosity (two bends > 70 degrees or one bend > 90 degrees), and circumflex CTO. Each variable could be assigned 1 point to grade the lesion difficulty [41]. Compared to the J-CTO score, previous failed PCI was not included in the PROGRESS-CTO score, as it could be operator-dependent. It is worth mentioning that all operators who contributed to PROGRESS-CTO score derivation were from high-volume CTO centers, with a high procedural success rate (92.9%). In a cohort of patients when experts and learning operators were involved, the predictive performance of the PROGRESS-CTO score was lower [37]. That is why PROGRESS-CTO score seems appropriate for operators from high-volume CTO centers, while in other conditions, its performance appears to be limited.

A more complex six-item score (previous CABG, age, tortuosity, calcification, stump, and occlusion length), CASTLE (EuroCTO), was derived and validated in a large cohort of patients. CASTLE score represents a valid alternative to the J-CTO score, with similar or even better discriminative power. However, the operators involved were from high-volume CTO centers, and the score might have limited value in other circumstances [39].

CT-derived scores represent an exciting field in successful PCI prediction for CTO. RECHARGE score was developed initially as an angiography-derived score; then, it was also validated as a CT-derived score. Six variables were incorporated in the final prediction model: lesion length (≥20 mm), calcification, blunt stump, tortuosity, diseased distal landing zone, and history of CABG on the target vessel. For each variable, 1 point could be assigned to highlight the lesion difficulty [28]. CT-derived RECHARGE score appeared to be a good predictor of PCI outcome and procedural time [27]. Other scores derived from CT examination, CT-RECTOR, and KCCT scores are potentially valuable tools in successful PCI prediction and could be extensively applied in clinical practice [27,34,43,44]. CT-RECTOR score consists of six variables: multiple occlusions, blunt stump, severe calcification, bending, previous failed PCI, and CTO duration [34]. KCCT score is more complex, as it includes seven variables: proximal blunt entry, proximal side branch, bending, occlusion length, peripheral calcification, central calcification, previous failed PCI, and CTO duration [44].

Even though the scores mentioned above had modest to good predictive value for PCI outcome prediction, a key variable represented by the operator’s skills could not be measured objectively and was not incorporated in the final prediction models. In general, experienced CTO operators are defined as those who performed > 300 PCI for CTO and > 50 PCI per year [48]. Thus, differences might occur when applying scores by operators with different experience levels.

## 5. Conclusions

Clinical prediction models are valuable tools for PCI success prediction in patients with CTO, even in the contemporary era with various techniques. As PCI for CTO is one of the most complex interventions, better preparation for the procedure and selecting the appropriate strategy are at the base of successful coronary recanalization. However, scores should be used complementary to clinical judgment, as no score assures a perfect predictive performance. Clinicians should be aware of the limitations of each model and should be able to correctly select the most appropriate score according to real-life case particularities such as lesion complexity and operator experience in order to maximize success and achieve the best patients’ outcomes.

## Figures and Tables

**Table 1:** General characteristics of studies included in the present systematic review.

Study, Year	Design	Patients, No	Age, Median/Mean	Setting	Scores Used	Outcomes	PCI Success, No (%)
Alessandrino et al., 2015 [15]	Observational, single-center	1143 (derivation cohort)	63.7 ± 11.5	First CTO-PCI attempt	CL J-CTO	Successful procedure	1202 (72.5)
		514 (validation cohort)	64.7 ± 11				
Chai et al., 2016 [16]	Observational, single-center, retrospective	152 (derivation cohort)	59.38 ± 10.29	CTO-PCI by retrograde approach (*n* = 228)	Original	Successful retrograde procedure	121 (79.6) derivation cohort
		76 (validation cohort)	59.28 ± 10.79				56 (73.6) validation cohort
Christopoulos et al., 2015 [17]	Observational, multicenter, retrospective	650	65 ± 10	CTO-PCI by retrograde and/or antegrade approach (*n* = 657)	J-CTO	Technical success	611 (93.0)
						Procedural success	601 (91.5)
Danek et al., 2016 [18]	Observational, multicenter, retrospective	1569	65 ± 10	First CTO-PCI	Original	Procedural complications	1380 (88)
Ellis et al., 2017 [19]	Observational, multicenter	291 (training cohort)	64 ± 10	CTO-PCI by hybrid approach (*n* = 456)	Original	Technical success	362 (79.4)
		145 (validation cohort)	62 ± 11				
Fujino et al., 2017 [20]	Observational, single-center, retrospective	205	69	CTO-PCI by retrograde and/or antegrade approach (*n* = 218)	J-CTO	Procedural success Guidewire crossing within 30 min	180 (82.6)
Galassi et al., 2016 [21]	Observational, single-center, retrospective	1019	61.1 ± 9.7	CTO-PCI performed by a single operator (*n* = 1073)	J-CTO ORA	Procedural success Technical failure	361 (87.8)–625 (94.4)
Kalnins et al., 2019 [22]	Observational, single-center, retrospective	551	63.5 ± 10.4	CTO-PCI performed by a single operator	J-CTO PROGRESS-CTO CL CASTLE (EuroCTO)	Procedural success	454 (82.4)
Kalogeropoulos et al., 2020 [23]	Observational, single-center, retrospective	660	65.8 ± 10.6	CTO-PCI by retrograde and/or antegrade approach	J-CTO CASTLE (EuroCTO)	Technical success	516 (78)
Karatasakis et al., 2016 [24]	Observational, multicenter, retrospective	658	66 ± 10	CTO-PCI by retrograde and/or antegrade approach (*n* = 664)	CL J-CTO PROGRESS-CTO	Technical success Procedural success	577 (87)
Khanna et al., 2018 [25]	Observational, single-center	285 (derivation cohort)	57.7 ± 9.5	CTO-PCI performed by single primary operator	W-CTO J-CTO	Technical success	341 (83.6)
		123 (validation cohort)	56.1 ± 9.3				
Li et al., 2015 [26]	Observational, retrospective	159	65.6 ± 11.9	CTO-PCI (*n* = 171)	J-CTO_CT_ J-CTO	Technical success	29 (52.7) in complex lesion
Li et al., 2021 [27]	Observational, single-center, retrospective	124	54	CTO-PCI by retrograde and/or antegrade approach (*n* = 131)	RECHARGE J-CTO CT-RECTOR KCCT	Procedural success Guidewire crossing within 30-min	94 (72)
Maeremans et al., 2017 [28]	Observational, multicenter, retrospective	590 (derivation cohort)	65 ± 11	CTO-PCI by hybrid approach	RECHARGE J-CTO PROGRESS-CTO	Technical success	490 (83) derivation cohort
		290 (validation cohort)	67 ± 11				247 (85) validation cohort
de Castro-Filho et al., 2017 [29]	Observational, single-center, retrospective	174	59.5	PCI for single CTO lesion by retrograde and/or antegrade approach	J-CTO	Technical failure	141 (81.0)
Morino et al., 2011 [30]	Observational, multicenter, retrospective	329 (derivation cohort)	≥75 (26.4%)	PCI for native coronary CTO lesion (*n* = 494)	J-CTO	Guidewire crossing within 30 min	238 (48.2)
		165 (validation cohort)	≥75 (27.3%)				
Nagamatsu et al., 2020 [31]	Observational, multicenter, retrospective	630	65.8 ± 10.7	CTO-PCI by retrograde approach (*n* = 886)	J-Channel	Collateral channel guidewire crossing success	531 (84.3)
Namazi et al., 2017 [32]	Observational, single-center, retrospective	183	59 ± 9	CTO-PCI by antegrade approach (*n* = 188)	Antegrade CTO	Procedural success and failure	121 (66.1)
Oktaviono et al., 2020 [33]	Observational, multicenter, retrospective	287	57 ± 8.6	CTO-PCI	Original	Successful PCI	205 (71.3)
Opolski et al., 2015 [34]	Observational, multicenter, retrospective	229	63 ± 10	CTO-PCI by retrograde and/or antegrade approach (*n* = 240)	CT-RECTOR J-CTO	Guidewire crossing within 30 min	123 (53.7)
Rigueira et al., 2020 [35]	Observational, single-center	334	68 ± 11	CTO-PCI by antegrade and/or retrograde approach (*n* = 377)	CTo-aBCDE	Successful PCI	228 (60.4)
Roller et al., 2016 [36]	Observational, single-center, retrospective	41	63.1 ± 8.3	CTO-PCI	Original (CT-derived)	Successful PCI	NA
Salinas et al., 2021 [37]	Observational, multicenter, retrospective	1342	65.17 ± 11.11	CTO-PCI performed by experts and learning operators	CASTLE (EuroCTO) J-CTO PROGRESS-CTO CL	Successful PCI	1044 (77.8)
Su et al., 2019 [38]	Observational, single-center, retrospective	246	79.43 ± 3.289 (elderly)	CTO-PCI in elderly patients (≥75 years)	J-CTO PROGRESS-CTO CL ORA	Procedural success	50 (73.53) elderly
			62.78 ± 8.478 (non-elderly)				151 (84.83) non-elderly
Szijgyarto et al., 2019 [39]	Observational, multicenter, retrospective	14,882 (derivation cohort)	Stratified in tertiles	CTO-PCI by retrograde and/or antegrade approach	CASTLE (EuroCTO)	Procedural success	12,526 (84.2)
		5745 (validation cohort)	64.2 ± 10.4				5042 (87.8)
Wilson et al., 2016 [40]	Observational, multicenter, retrospective	1156	65.2 ± 10.2	CTO-PCI performed by hybrid approach	J-CTO Original	Procedural success Guidewire crossing within 30 min	912 (79) first attempt
							1037 (90) overall
Christopoulos et al., 2016 [41]	Observational, multicenter, retrospective	521 (derivation cohort)	65 ± 10	CTO-PCI (*n* = 781)	PROGRESS-CTO J-CTO	Technical success	726 (92.9)
		260 (validation cohort)	66 ± 10				
Huang et al., 2018 [42]	Observational, single-center, retrospective	216	61.6 ± 11.3	CTO-PCI performed by retrograde approach	Original	Technical success	197 (91.2)
Tan et al., 2017 [43]	Observational, single-center, retrospective	191	61 ± 11	CTO-PCI by retrograde and/or antegrade approach	CT-RECTOR J-CTO	Guidewire crossing within 30 min Procedural success	145 (76)
Yu et al., 2017 [44]	Observational, multicenter, retrospective	643	62	CTO-PCI by retrograde and/or antegrade approach (*n* = 684)	KCCT J-CTO PROGRESS-CTO CL CT-RECTOR	Guidewire crossing within 30 min Procedural success	479 (74)
Jin et al., 2017 [45]	Observational, single-center, retrospective	438	61	CTO-PCI by retrograde and/or antegrade approach	Busan CTO J-CTO	Successful PCI	355 (81.1)
Gong et al., 2021 [46]	Observational, multicenter, retrospective	402 (derivation cohort)	61.1 ± 10.1	PCI by antegrade approach for in-stent chronic total occlusion	IS-CTO J-CTO PROGRESS-CTO	Successful PCI	367 (77.4)
		72 (validation cohort)	59.3 ± 9.1				

CASTLE—coronary artery bypass grafting history, age (≥70 years), stump anatomy, tortuosity degree, length of occlusion, and extent of calcification score; CL—clinical and lesion-related score; CT-RECTOR—computed tomography registry of chronic total occlusion revascularization score; CTO—coronary chronic total occlusion; IS-CTO—in-stent chronic total occlusion score; J-CTO—Japanese chronic total occlusion score; KCCT—Korean multicenter CTO CT registry score; ORA—ostial location, Rentrop grade, age score; PCI—percutaneous coronary intervention; PROGRESS-CTO—prospective global registry for the study of chronic total occlusion intervention score; RECHARGE—coronary CT angiograph-derived registry of crossboss and hybrid procedures in France, the Netherlands, Belgium, and United Kingdom score; W-CTO—weighted chronic total occlusion score.

**Table 2:** Results reported in studies included in the present systematic review.

Study, Year	Scores	Results	
		c-Statistic/AUC	*p*-Value
Alessandrino et al., 2015 [15]	CL (validation cohort)	0.68 (95% CI, 0.63–0.73)	
	J-CTO (validation cohort)	0.60 (95% CI, 0.54–0.65)	
Chai et al., 2016 [16]	Original (derivation cohort)	0.832 ± 0.042	
	Original (validation cohort)	0.912 ± 0.041	
Christopoulos et al., 2015 [17]	J-CTO	0.705	
Danek et al., 2016 [18]	Original—derivation cohort	0.758 (95% CI, 0.665–0.850)	
	Original—validation cohort	0.793 (95% CI, 0.682–0.905)	
Ellis et al., 2017 [19]	Original (7-item model)—training cohort	0.753	
	Original (7-item model)—validation cohort	0.738	
	J-CTO	0.55	
	PROGRESS CTO	0.61	
Fujino et al., 2017 [20]	J-CTO CT-derived (procedural success)	0.855 (95% CI, 0.797–0.912)	
	J-CTO angiography-derived (procedural success)	0.698 (95% CI, 0.615–0.782)	
	J-CTO CT-derived (30-min wire crossing)	0.812 (95% CI, 0.752–0.871)	
	J-CTO angiography-derived (30-min wire crossing)	0.692 (95% CI, 0.621–0.764)	
Galassi et al., 2016 [21]	J-CTO (technical failure)	0.556	*p* = 0.05
	ORA (derivation cohort)	0.728 (95% CI, 0.652–0.804)	
	ORA (validation cohort)	0.772 (95% CI, 0.657–0.887)	
Kalnins et al., 2019 [22]	J-CTO	0.714 (95% CI, 0.660–0.768)	*p* < 0.001
	PROGRESS-CTO	0.605 (95% CI, 0.546–0.665)	*p* = 0.001
	CASTLE	0.641 (95% CI, 0.581–0.701)	*p* < 0.001
	CL	0.624 (95% CI, 0.565–0.683)	*p* < 0.001
Kalogeropoulos et al., 2020 [23]	J-CTO (whole cohort)	0.694 (95% CI, 0.649–0.739)	*p* < 0.001
	J-CTO (complex lesion, score ≥ 3)	0.473 (95% CI, 0.393–0.553)	*p* < 0.52
	CASTLE (whole cohort)	0.674 (95% CI, 0.625–0.724)	*p* < 0.001
	CASTLE (complex lesion, score ≥ 4)	0.588 (95% CI, 0.509–0.668)	*p* = 0.03
Karatasakis et al., 2016 [24]	CL	0.691 (95% CI, 0.633–0.749)	*p* < 0.001
	J-CTO	0.682 (95% CI, 0.625–0.738)	*p* < 0.001
	PROGRESS-CTO	0.647 (95% CI, 0.588–0.706)	*p* < 0.001
	CL (antegrade procedures)	0.746 (95% CI, 0.663–0.829)	*p* < 0.001
	J-CTO (antegrade procedures)	0.735 (95% CI, 0.650–0.821)	*p* < 0.001
	PROGRESS-CTO (antegrade procedures)	0.692 (95% CI, 0.610–0.774)	*p* < 0.001
Khanna et al., 2018 [25]	W-CTO	0.86	
	J-CTO	0.82	
Li et al., 2015 [26]	J-CTO (CT-derived score)	0.882 (95% CI, 0.824–0.927)	*p* < 0.001
	J-CTO (angiography-derived score)	0.868 (95% CI, 0.808–0.915)	*p* < 0.001
Li et al., 2021 [27]	Procedural success		
	RECHARGE (CT-derived)	0.718 (95% CI, 0.633–0.793)	
	RECHARGE (angiography derived)	0.757 (95% CI, 0.661–0.840)	
	J-CTO (CT-derived)	0.704 (95% CI, 0.618–0.780)	
	CT-RECTOR	0.665 (95% CI, 0.577–0.745)	
	KCCT	0.717 (95% CI, 0.631–0.792)	
	30-min wire crossing		
	RECHARGE (CT-derived)	0.708 (95% CI, 0.622–0.784)	
	RECHARGE (angiography derived)	0.705 (95% CI, 0.603–0.793)	
	J-CTO (CT-derived)	0.673 (95% CI, 0.586–0.752)	
	CT-RECTOR	0.643 (95% CI, 0.544–0.724)	
	KCCT	0.703 (95% CI, 0.617–0.780)	
Maeremans et al., 2017 [28]	RECHARGE—derivation cohort	0.783 (95% CI, 0.74–0.83)	
	RECHARGE—validation cohort	0.711 (95% CI, 0.63–0.79)	
	J-CTO	0.676 (95% CI, 0.59–0.76)	
	PROGRESS-CTO	0.608 (95% CI, 0.52–0.70)	
Castro-Filho et al., 2017 [29]	J-CTO (occlusion < 12 months)	0.766	
	J-CTO (occlusion ≥ 12 months)	0.705	
	J-CTO (indeterminate duration occlusion)	0.798	
Morino et al., 2011 [30]	J-CTO (derivation cohort)	0.82	
	J-CTO (validation cohort)	0.76	
Nagamatsu et al., 2020 [31]	Septal collateral channel set		
	J-Channel (derivation cohort)	0.744	
	J-Channel (validation cohort)	0.743	
	Non-septal collateral channel set		
	J-Channel (derivation cohort)	0.757	
	J-Channel (validation cohort)	0.826	
Namazi et al., 2017 [32]	Antegrade CTO (PCI failure)	0.839 (95% CI, 0.778–0.9)	
Oktaviono et al., 2020 [33]	Original (5-item model)	0.89	
Opolski et al., 2015 [34]	CT-RECTOR	0.83	
	J-CTO	0.71	
Rigueira et al., 2020 [35]	CTo-aBCDE	0.831	
Roller et al., 2016 [36]	Original (5-item model)	0.8232	
Salinas et al., 2021 [37]	CASTLE	0.633 (95% CI, 0.60–0.67)	
	J-CTO	0.628 (95% CI, 0.59–0.67)	
	PROGRESS-CTO	0.557 (95% CI, 0.52–0.59)	
	CL	0.652 (95% CI, 0.62–0.69)	
Su et al., 2019 [38]	Elderly patients		
	J-CTO	0.791 (95% CI, 0.688–0.894)	
	PROGRESS-CTO	0.788 (95% CI, 0.684–0.893)	
	CL	0.711 (95% CI, 0.576–0.845)	
	ORA	0.703 (95% CI, 0.573–0.834)	
	All patients		
	J-CTO	0.806 (95% CI, 0.753–0.859)	*p* < 0.0001
	PROGRESS-CTO	0.727 (95% CI, 0.656–0.799)	*p* < 0.0001
	CL	0.800 (95% CI, 0.737–0.863)	*p* < 0.0001
	ORA	0.672 (95% CI, 0.587–0.757)	*p* < 0.0001
Szijgyarto et al., 2019 [39]	CASTLE (derivation cohort)	0.66	
	CASTLE (validation cohort)	0.68	
	J-CTO (derivation cohort)	0.63	
	J-CTO (validation cohort)	0.64	
Wilson et al., 2016 [40]	J-CTO	0.68 (95% CI, 0.64–0.71)	*p* < 0.001
	Original	0.72 (95% CI, 0.68–0.75)	*p* < 0.001
	J-CTO (wiring time within 30 min)	0.79 (95% CI, 0.76–0.82)	
Christopoulos et al., 2016 [41]	PROGRESS-CTO (derivation cohort)	0.778	
	PROGRESS-CTO (validation cohort)	0.720	
	J-CTO (validation cohort)	0.746	
Huang et al., 2018 [42]	Original (collateral channel tracking)	0.800	
	Original (technical success)	0.752	
Tan et al., 2017 [43]	CT-RECTOR (wiring within 30 min)	0.85	
	J-CTO (wiring withing 30 min)	0.76	
Yu et al., 2017 [44]	Successful guidewire crossing within 30 min (derivation cohort)		
	KCCT	0.776 (95% CI, 0.735–0.818)	
	J-CTO	0.714 (95% CI, 0.669–0.758)	
	PROGRESS-CTO	0.651 (95% CI, 0.504–0.700)	
	CL	0.682 (95% CI, 0.624–0.730)	
	CT-RECTOR	0.718 (95% CI, 0.674–0.763)	
	Final procedural success (derivation cohort)		
	KCCT	0.773 (95% CI, 0.728–0.819)	
	J-CTO	0.672 (95% CI, 0.620–0.724)	
	PROGRESS-CTO	0.558 (95% CI, 0.616–0.720)	
	CL	0.658 (95% CI, 0.602–0.713)	
	CT-RECTOR	0.708 (95% CI, 0.658–0.758)	
Jin et al., 2017 [45]	Busan CTO	0.681	
	J-CTO	0.598	
Gong et al., 2021 [46]	IS-CTO	0.976	
	PROGRESS-CTO	0.579	
	J-CTO	0.642	

AUC—area under the curve; CASTLE—coronary artery bypass grafting history, age (≥ 70 years), stump anatomy, tortuosity degree, length of occlusion, and extent of calcification score; CL—clinical and lesion-related score; CT-RECTOR—computed tomography registry of chronic total occlusion revascularization score; CTO—coronary chronic total occlusion; IS-CTO—in-stent chronic total occlusion score; J-CTO—Japanese chronic total occlusion score; KCCT—Korean multicenter CTO CT registry score; ORA—ostial location, Rentrop grade, age score; PCI—percutaneous coronary intervention; PROGRESS-CTO—prospective global registry for the study of chronic total occlusion intervention score; RECHARGE—coronary CT angiograph-derived registry of crossboss and hybrid procedures in France, the Netherlands, Belgium, and United Kingdom score; W-CTO—weighted chronic total occlusion score.

## Supplementary Materials

The following are available online at https://www.mdpi.com/article/10.3390/healthcare9081033/s1, Table S1: Databases and search strategies used in present systematic review; Table S2: Risk of bias and applicability assessment using PROBAST tool. Table S3. Scores and variables for successful PCI prediction and their performance reported in studies.
