# Supplementary material for: Procedural Success Prediction Scoring Systems Used in Percutaneous Coronary Interventions for Chronic Total Occlusions: A Systematic Evaluation
Source: Healthcare (Basel). 2021 Aug 11;9(8):1033. doi: 10.3390/healthcare9081033 (PMC8393835; doi:10.3390/healthcare9081033)
Supplement: Supplementary file 1 [file healthcare-09-01033-s001.zip › Table S1 Search Strategy.pdf]

Table S1. Databases and search strategies used in present systematic review

Database	Coverage	Search run	Records
**MEDLINE**	1946 to present	"(percutaneous coronary intervention) AND (chronic total occlusion)"	163
		"((coronary chronic total occlusion) AND (prediction)) AND (score)"	9
		"(J-CTO) AND (coronary chronic total occlusion)"	12
		"(PROGRESS-CTO) AND (coronary chronic total occlusion)"	1
		"castle AND coronary AND chronic AND total AND occlusion"	1
		"((CL) AND (Score)) AND (coronary chronic total occlusion)"	4
		"((ORA) AND (score)) AND (coronary chronic total occlusion)"	0
		"(RECHARGE) AND (coronary chronic total occlusion)"	0
		"(CT-RECTOR) AND (coronary chronic total occlusion)"	0
		"(KCCT) AND (coronary chronic total occlusion)"	0
			Total records: 190
**Embase**	1966 to present	"percutaneous AND coronary AND intervention AND chronic AND total AND occlusion"	112
		"coronary AND chronic AND total AND occlusion AND prediction AND score"	80
		"'j-cto' AND coronary AND chronic AND total AND occlusion"	403
		"'progress cto' AND coronary AND chronic AND total AND occlusion"	84
		"castle AND coronary AND chronic AND total AND occlusion"	26
		"cl AND score AND coronary AND chronic AND total AND occlusion"	15
		"ora AND score AND coronary AND chronic AND total AND occlusion"	3
		"recharge AND coronary AND chronic AND total AND occlusion"	19
		"'ct rector' AND coronary AND chronic AND total AND occlusion"	6
		"kcct AND coronary AND chronic AND total AND occlusion"	2
			Total records: 750
**Cochrane library**	1967 to present	"percutaneous AND coronary AND intervention AND chronic AND total AND occlusion"	236
		"coronary AND chronic AND total AND occlusion AND prediction AND score"	21
		"j-cto AND coronary AND chronic AND total AND occlusion"	29
		"progress-cto AND coronary AND chronic AND total AND occlusion"	5
		"castle AND coronary AND chronic AND total AND occlusion"	1
		"cl AND score AND coronary AND chronic AND total AND occlusion"	2
		"ora AND score AND coronary AND chronic AND total AND occlusion"	1
		"recharge AND coronary AND chronic AND total AND occlusion"	1

	“'ct rector' AND coronary AND chronic AND total AND occlusion”	2
	“kcct AND coronary AND chronic AND total AND occlusion”	1
		Total records 299
