# Supplementary material for: Procedural Success Prediction Scoring Systems Used in Percutaneous Coronary Interventions for Chronic Total Occlusions: A Systematic Evaluation
Source: Healthcare (Basel). 2021 Aug 11;9(8):1033. doi: 10.3390/healthcare9081033 (PMC8393835; doi:10.3390/healthcare9081033)
Supplement: Supplementary file 1 [file healthcare-09-01033-s001.zip › Table S2 Risk of Bias.pdf]

Table S2. Risk of bias and applicability assessment using PROBAST tool.

Study	ROB*				Applicability (AP)			Overall	
	PRT**	PRD***	Outcome	Analysis	PRT	PRD	Outcome	ROB	AP
Alessandrino, 2015	+	?	?	-	+	+	+	-	+
Chai, 2016	+	?	?	-	+	+	+	-	+
Christopoulos, 2015	+	?	?	?	+	+	+	?	+
Danek, 2016	+	?	?	?	+	+	+	?	+
Ellis, 2017	+	+	?	-	+	+	+	-	+
Fujino, 2017	+	+	?	+	+	+	+	?	+
Galassi, 2016	+	?	?	+	+	+	+	?	+
Kalnins, 2019	+	?	?	-	+	+	+	-	+
Kalogeropoulos, 2020	+	?	?	+	+	+	+	?	+
Karatasakis, 2020	+	?	?	?	+	+	+	?	+
Khanna, 2018	+	?	?	?	+	+	+	?	+
Li, 2015	+	+	+	?	+	+	+	?	+
Li, 2021	+	+	?	-	+	+	+	-	+
Maeremans, 2017	+	?	?	?	+	+	+	?	+
Castro-Filho, 2017	+	?	?	?	+	+	+	?	+
Morino, 2011	+	?	?	-	+	+	+	-	+
Nagamatsu, 2020	+	?	?	-	+	+	+	-	+
Namazi, 2017	+	?	?	?	+	+	+	?	+
Oktaviono, 2020	+	+	+	?	+	+	+	?	+
Opolski, 2015	+	+	+	-	+	+	+	-	+
Rigueira, 2020	+	?	?	-	+	+	+	-	+
Roller, 2016	+	?	?	-	+	+	+	-	+
Salinas, 2021	+	?	?	?	+	+	+	?	+
Su, 2019	+	?	?	-	+	+	+	-	+
Szijgyarto, 2019	+	?	?	+	+	+	+	?	+
Wilson, 2016	+	?	?	?	+	+	+	?	+
Christopoulos, 2016	+	?	?	?	+	+	+	?	+
Huang, 2018	+	?	?	-	+	+	+	-	+
Tan, 2017	+	+	+	?	+	+	+	?	+
Yu, 2017	+	+	?	-	+	+	+	-	+
Jin, 2017	+	?	?	?	+	+	+	?	+
Gong, 2021	+	?	?	?	+	+	+	?	+

ROB* = risk of bias; PRT** = participants; PRD*** = predictors;
+ indicates low ROB/low concern regarding applicability; - indicates high ROB/concern regarding applicability; ? indicates unclear ROB/unclear concern regarding applicability
