# Supplementary material for: Procedural Success Prediction Scoring Systems Used in Percutaneous Coronary Interventions for Chronic Total Occlusions: A Systematic Evaluation
Source: Healthcare (Basel). 2021 Aug 11;9(8):1033. doi: 10.3390/healthcare9081033 (PMC8393835; doi:10.3390/healthcare9081033)
Supplement: Supplementary file 1 [file healthcare-09-01033-s001.zip › Table S3.pdf]

Table S3. Scores and variables for successful PCI prediction and their performance reported in studies.

Scores	J-CTO	PROGRESS-CTO	CL	CASTLE	KCCT
**Variables**	Previously failed lesion	Proximal cap ambiguity	Severe calcified lesion	Previous CABG	Blunt proximal entry site
	Blunt stump type	Absence of “interventional” collaterals	Previous CABG	Age	Proximal adjacent side branch
	Bending	Moderate/severe tortuosity	Lesion length ≥ 20 mm	Tortuosity	Occlusion length ≥ 15 mm
	Calcification	Circumflex CTO	Previous MI	Calcification	Bend > 45 degree
	Occlusion length ≥ 20 mm		Blunt stump	Stump	Severe calcification
			Non-LAD CTO location	Length of CTO	Previously failed CTO PCI
					Occlusion duration ≥ 12 month or unknown
**AUC**	0.55 – 0.868	0.557 – 0.788	0.624 – 0.800	0.633 – 0.68	0.703 – 0.776

AUC – area under the curve; CASTLE – coronary artery bypass grafting history, age (≥ 70 years), stump anatomy, tortuosity degree, length of occlusion and extent of calcification score; CL – clinical and lesion-related score; CTO – coronary chronic total occlusion; KCCT – Korean multicenter CTO CT registry score; PCI – percutaneous coronary intervention; PROGRESS-CTO - prospective global registry for the study of chronic total occlusion intervention score.
